# A comprehensive RNA-Seq-based gene expression atlas of the summer squash (*Cucurbita pepo)* provides insights into fruit morphology and ripening mechanisms

**DOI:** 10.1186/s12864-021-07683-2

**Published:** 2021-05-12

**Authors:** Aliki Xanthopoulou, Javier Montero-Pau, Belén Picó, Panagiotis Boumpas, Eleni Tsaliki, Harry S. Paris, Athanasios Tsaftaris, Apostolos Kalivas, Ifigeneia Mellidou, Ioannis Ganopoulos

**Affiliations:** 1 Institute of Plant Breeding and Genetic Resources, Hellenic Agricultural Organization DIMITRA (ex NAGREF), GR-57001 Thermi, Macedonia Greece; 2 Cavanilles Institute of Biodiversity and Evolutionary Biology (ICBiBE), Universitat de València, 46022 Valencia, Spain; 3 Institute for the Conservation and Breeding of Agricultural Biodiversity (COMAV-UPV), Universitat Politècnica de València, Camino de Vera s/n, 46022 Valencia, Spain; 4 Department of Vegetable Crops and Plant Genetics, Agricultural Research Organization, Newe Ya‘ar Research Center, 3009500 Ramat Yishay, Israel; 5 Perrotis College, American Farm School, GR-57001 Thessaloniki, Greece

**Keywords:** Gene expression atlas, *Cucurbita pepo*, RNA-seq, Differential gene expression, Plant growth and development, *Cucurbitaceae*, Novel genes, Fruit growth and ripening

## Abstract

**Background:**

Summer squash (*Cucurbita pepo*: *Cucurbitaceae*) are a popular horticultural crop for which there is insufficient genomic and transcriptomic information. Gene expression atlases are crucial for the identification of genes expressed in different tissues at various plant developmental stages. Here, we present the first comprehensive gene expression atlas for a summer squash cultivar, including transcripts obtained from seeds, shoots, leaf stem, young and developed leaves, male and female flowers, fruits of seven developmental stages, as well as primary and lateral roots.

**Results:**

In total, 27,868 genes and 2352 novel transcripts were annotated from these 16 tissues, with over 18,000 genes common to all tissue groups. Of these, 3812 were identified as housekeeping genes, half of which assigned to known gene ontologies. Flowers, seeds, and young fruits had the largest number of specific genes, whilst intermediate-age fruits the fewest. There also were genes that were differentially expressed in the various tissues, the male flower being the tissue with the most differentially expressed genes in pair-wise comparisons with the remaining tissues, and the leaf stem the least. The largest expression change during fruit development was early on, from female flower to fruit two days after pollination. A weighted correlation network analysis performed on the global gene expression dataset assigned 25,413 genes to 24 coexpression groups, and some of these groups exhibited strong tissue specificity.

**Conclusions:**

These findings enrich our understanding about the transcriptomic events associated with summer squash development and ripening. This comprehensive gene expression atlas is expected not only to provide a global view of gene expression patterns in all major tissues in *C. pepo* but to also serve as a valuable resource for functional genomics and gene discovery in *Cucurbitaceae*.

**Supplementary Information:**

The online version contains supplementary material available at 10.1186/s12864-021-07683-2.

## Background

Summer squash are the tender, young fruits of *Cucurbita pepo* L. (*Cucurbitaceae*). *C. pepo* is an extremely polymorphic species that is considered to consist of eight edible-fruited cultivar-groups or morphotypes, based on differences in fruit shape [1]. Cultivars of six of these morphotypes, namely Cocozelle, Crookneck, Scallop, Straightneck, Vegetable Marrow, and Zucchini, have a fruit shape that deviates markedly from the 1:1 length-to-width ratio, and the cultivars of these groups are grown for their summer squash. Besides the marked differences in fruit shape, the very numerous cultivars of summer squash also display a broad range of diversity in flowering and fruit traits [2].

During the last decade, the development of novel genomic technologies such as next-generation sequencing and other high-throughput technologies, have been widely applied with the goal of obtaining novel insights on gene expression data and plant responses to stress [3, 4]. In order to achieve genotype-phenotype association, combinations of genome-wide data and gene expression profiles for different developmental stages of summer squash development is of utmost importance. Gene expression atlases are crucial for the identification of genes expressed in different tissues at various plant developmental stages.

Despite the fact that summer squash is a popular, high-value horticultural crop, relatively little genomic and transcriptomic data are available for it so far. Research efforts with -omics of summer squash include genome assembly [5, 6], transcriptome development [7, 8], and SNP-based genetic maps developed from the cross between subsp. *pepo* Zucchini × subsp. *ovifera* Scallop [9, 10]. Whole-genome sequencing of the Zucchini accession ‘BGV004370’ is the first reference genome of summer squash [10]. A draft of the ‘True French’ Zucchini proteome is available [11], while RNA-seq technologies have been employed to study zucchini parthenocarpy [12].

The objective of the present study was to develop a Gene Expression Atlas (CupeGEA) for the *C. pepo* subsp. *pepo* summer squash ‘Kompokolokytho’ based on 16 vegetative and fruit tissues during development and ripening. This gene expression atlas of squash is expected not only to provide a global view of gene expression patterns in all major tissues and fruit developmental stages in *C. pepo* but to also serve as a valuable resource for functional genomics accelerating gene discovery in the *Cucurbitaceae*.

## Results and discussion

### RNA sequencing and read assembly

**Fig. 1:**
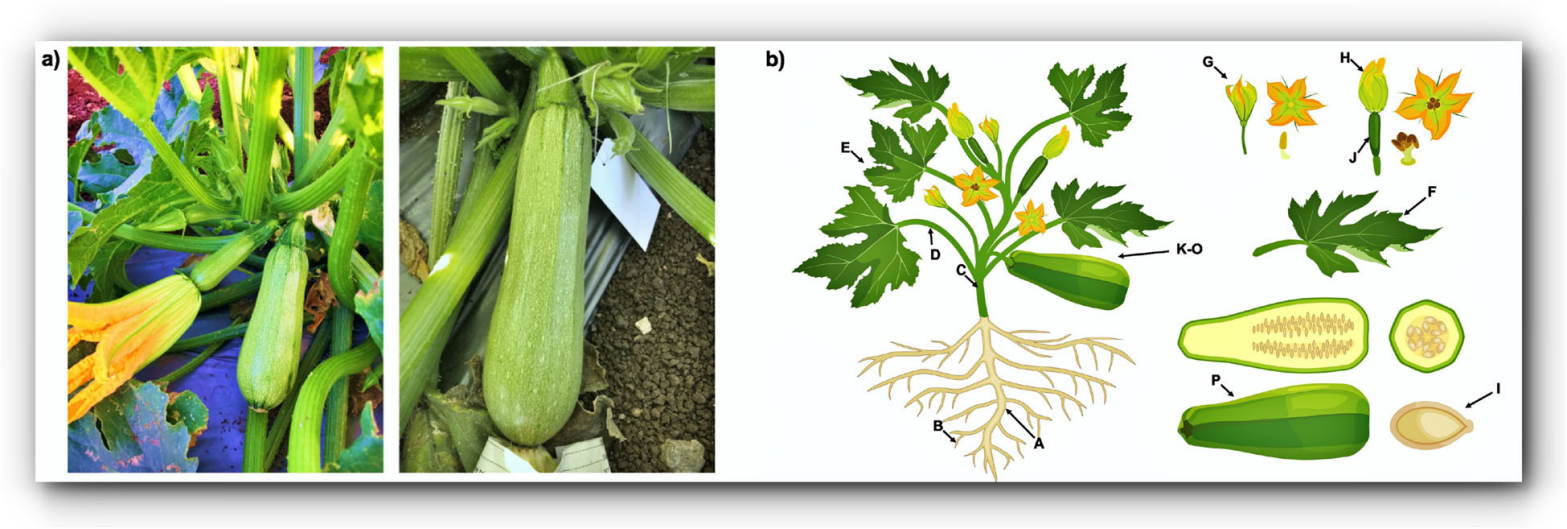
**a**. Left, plant of ‘Kompokolokytho’ summer squash. Note its bush growth habit, dark stem, spiculate petioles, unusually large pistillate-flower corolla, and the initial young fruit of light-medium green having vegetable marrow (short, tapered cylindrical) shape; right, close-up view of older ‘Kompokolokytho’ plant. Note the basal braching and the young fruit of light-medium green having cocozelle (long, bulbous cylindrical) shape. **b** Artist’s rendition of ‘Kompokolokytho’ summer squash indicating schematically the 16 plant tissues sampled for the RNA-seq atlas. A = primary root, B = lateral root, C = shoot, D = stem of leaves, E = young leaf, F = fully developed leaf, G = male flower, H = female flower, I = seed, J–P = eight developmental stages of fruit [2DAP (days after pollination); 7DAP; 10DAP; 15DAP; 20DAP; 30DAP; 40DAP-ripe fruit]

The 16 cDNA libraries from the various tissues, including primary and lateral roots, shoot, leaf stem, young and developed leaf, male and female flower, fruit in seven developmental stages and seed (Fig. 1), were analyzed on the BGISEQ-500 sequencing platform. After removing adapter sequences and low quality reads, an average of 82,900 M clean reads with a Q30 percentage ≥ 86% were generated per tissue (Table S1). The clean reads were mapped to the reference genome (*C. pepo* Genome v4.1) [5]. After removing rRNA (0.50 to 8.89%) and filter reads, the remaining reads of the various tissues were mapped. Mapping ratio ranged from 71.21% (lateral root) to 89.95% (young leaf), with an average of 84.68%.

The de novo transcriptome assembly allowed the identification of 665,782 transcripts from the 16 tissues (Table 1). The percentage of the clean reads that mapped against this new assembly, ranged from 84.52% (young leaf) to 65.70% (lateral root), whilst the uniquely mapping ratio varied from 61.29 (10DAP fruit) to 50.31% (lateral root). Total transcripts of each tissue varied from 42,429 to 45,239, of which the novel transcripts ranged from 25,093 to 26,870, known genes from 24,355 to 25,197, and novel genes from 1662 to 1829. These numbers are similar to those reported in transcriptome studies with melon [13], with pumpkin [14], and with winter squash, *C. pepo* Acorn morphotype [15].

**Table 1:** Statistics of the de novo transcriptome assembly and mapping of clean reads against the new transcriptome assembly including novel transcripts

Sample	No. Transcripts	Mapping Ratio (%)	Uniquely Mapping Ratio (%)	No. Novel Transcripts	No. genes	No. novel Genes
Shoot	42,429	79.03	60.09	25,093	26,017	1662
Leaf stem	44,242	79.35	59.25	26,305	26,545	1747
Young leaf	43,356	84.52	60.56	25,840	26,322	1669
Developed leaf	43,265	78.08	57.62	25,709	26,261	1706
Male flower	43,179	78.42	60.45	25,480	26,572	1744
Female flower	44,907	80.29	58.45	26,720	27,026	1829
2DAP fruit	45,239	80.06	60.57	26,786	26,795	1749
7DAP fruit	44,614	80.91	60.77	26,474	26,673	1721
10DAP fruit	44,833	81.44	61.29	26,799	26,736	1760
15DAP fruit	45,163	80.92	60.77	26,870	26,658	1744
20DAP fruit	44,420	81.24	61.08	26,511	26,556	1737
30DAP fruit	44,588	79.41	60.39	26,548	26,519	1734
Ripe fruit	43,859	79.52	59.24	26,079	26,391	1699
Seed	44,235	76.78	59.44	26,186	26,469	1708
Primary root	43,727	69.90	53.36	25,863	26,634	1738
Lateral root	43,704	65.70	50.31	25,855	26,582	1728

### Global gene expression patterns

**Fig. 2:**
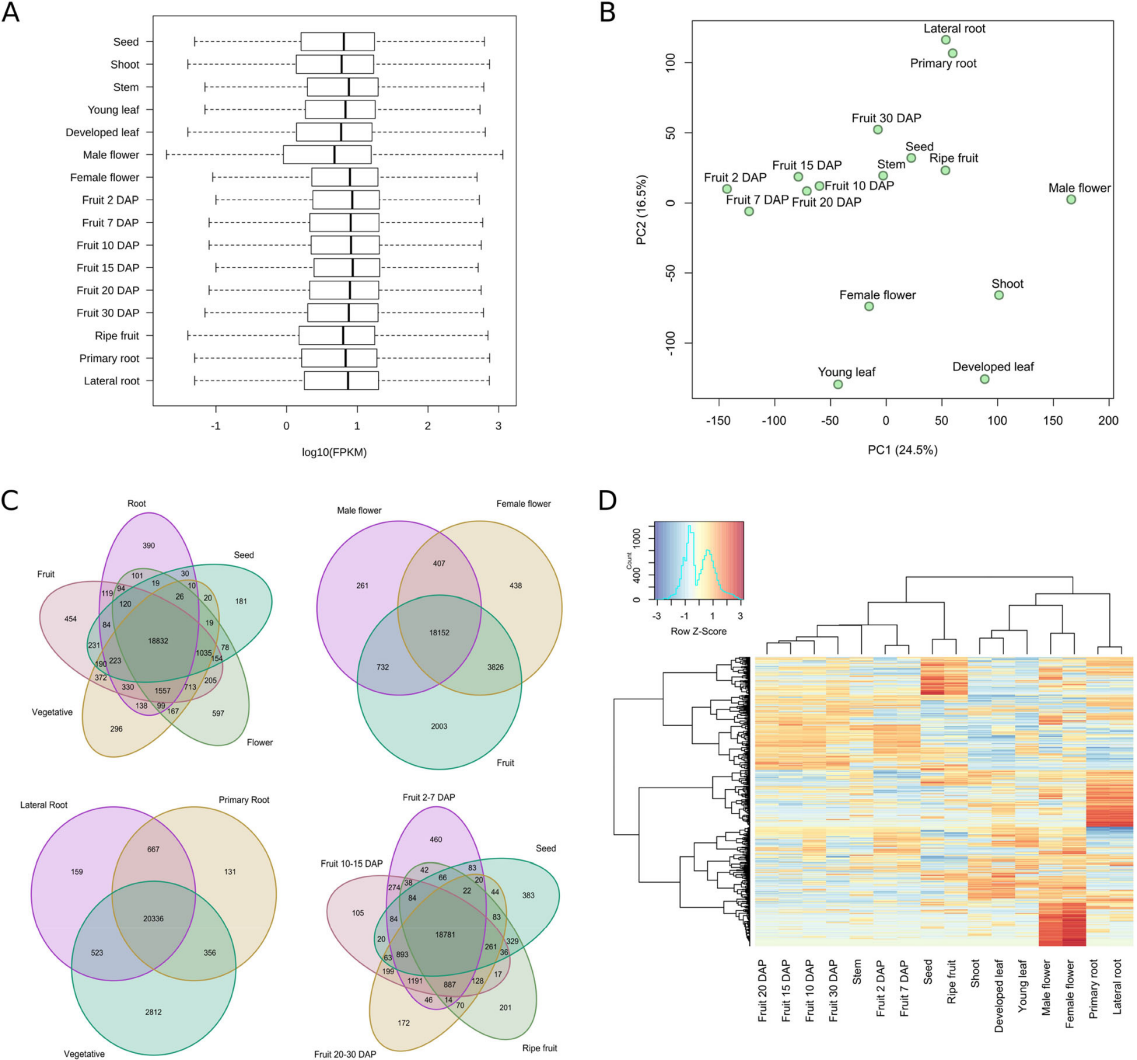
**a** Violin plot of the distribution of the gene expression in tissues. **b** Principal component analysis based on the expression levels of the various tissues. **c** Venn diagram showing the number of shared expressed genes (FPKM > 1) between different tissues or groups of tissues. Flower: female flower + male flower; Fruit at 2DAP, 7DAP, 10DAP, 15DAP, 20DAP, 30DAP, and Ripe fruit (40DAP); Root: lateral root and primary root; Vegetative: developed leaf, young leaf, stem and shoot. **d** Heatmap of the top 1000 genes with the highest expression variability. The color key represents normalized log_2_ FPKM. The top dendrogram shows the relationships among tissues and the side dendrogram relationships among genes

Of the total 27,868 annotated genes plus the 2352 novel genes, 26,895 had > 1 FPKM values for at least one tissue. Figure 2a shows the number of genes with different log10 FPKM values in the various tissues, displaying similar global expression levels. The expression of these genes was subjected to a Principal component analysis (PCA) (Fig. 2b). The 16 tissues are easily distinguished in the PCA. The first component, explaining 23.8% of the variation, shows a gradient separation of the fruit expression profiles, from early fruit developmental stages to late stages, indicating differences in gene expression over the course of fruit development. The seed profile was similar to that of the mature fruit. The second component, which explains 15.6% of the variation, separates the fruit profiles from those of the roots, which group near the top, and from those of the flowers, leaves and shoots, which are dispersed near the bottom. Clearly, some tissues have expression patterns more similar to others, with the early and intermediate fruit stages distinct from foliar and root tissues. Furthermore, root, the foliar, and flower tissues are well-separated, indicating differences among them in their gene expression profiles.

The first Venn diagram compares root tissues, fruit stages, vegetative tissues, flowers, and seeds (Fig. 2c). A total of 20,425 expressed genes were common to all these tissue groups, which is 88, 80, 82, 82, and 90% of the total number expressed in roots, fruits, vegetative tissues (shoot, leaf stem, and leaves), flowers, and seeds, respectively. Similar to other gene expression atlas [16, 17], transcriptional profiles were variable and diverse among the various tissues. In particular, seed and fruit tissues were, respectively, the ones that shared the highest and the lowest percentage of genes with the remaining tissues. Male and female flower tissues shared 20,982 expressed genes with each other, which were 94 and 88% of the total number of genes expressed in male and female flowers, respectively, of which 98% (20,509) were also shared with fruit tissues. Primary and lateral roots shared 22,246 expressed genes, which were 98 and 97% of the total number of genes expressed in primary and lateral roots, respectively, and 97% of these common genes (21,688) were also expressed in vegetative tissues. Fruit tissues shared 20,307 of their expressed genes, 85% of the genes expressed during early and intermediate fruit development (2DAP to 30DAP) and a 91% of the genes expressed in the ripe fruit. The number of shared genes dropped as the fruits developed, reflecting the dramatic transcriptome changes occurring during the ripening process, probably attributable to induction of metabolic pathways related to fruit aroma, taste and carotenoid composition, or the decline of photosynthetic activity [17].

Figure 2d represents a heat map and a dendrogram of the FPKM normalized log2-transformed generated with the 1000 more variable genes. The clustering of the transcriptional profiles of these highly variable genes suggests that there are two main groups of tissues, one including all the fruit tissues and the seeds, and the second the foliar, flower and root tissues. Within the fruit cluster, the ripe fruit grouped with the seeds, and the fruit developmental stages separated into two sub-groups, early (2DAP and 7DAP) and intermediate (10DAP to 30DAP). Within the other cluster, roots were separated from foliar and flower tissues, with separate sub-clusters each for foliage and flowers. This clustering is likely a result of the use of the more variable genes that are probably more specific in each tissue.

### Housekeeping genes

Housekeeping genes (HKG) are genes that show little variation across tissues, being expressed in all tissues and showing similar expression levels across them. A total of 3812 genes had stable expression over the 16 tissues, and thus considered as HKGs (Table S2), with 1650 of them assigned to a known gene ontology (GO). This is a number similar to the estimated number in humans [18], but a bit lower than that reported in other crops, such as olive tree (*Olea europaea* L.), which is thought to be of polyploid origin resulting in a high number of paralogues [19]. The enrichment analysis indicated a number of biological processes (BP) essential for cell function which are over-represented as compared with all expressed genes, including intracellular protein transport, vesicle-mediated transport, ubiquitin-dependent protein catabolic process, protein deubiquitination, mRNA splicing, protein transport, and the corresponding molecular functions (MF), such as RNA binding, translation initiation factor activity, GTP binding, ubiquitin protein ligase binding, and protein transporter activity. The Kyoto Encyclopedia of Genes and Genomes (KEGG) analysis also revealed a wide range of pathways, most representing genes involved in metabolism and biosynthesis. These genes can be further used in expression analysis to normalize the expression of other analyzed genes that are specific of tissue, developmental stage, or expressed under specific stimuli.

### Tissue-specific genes

**Fig. 3:**
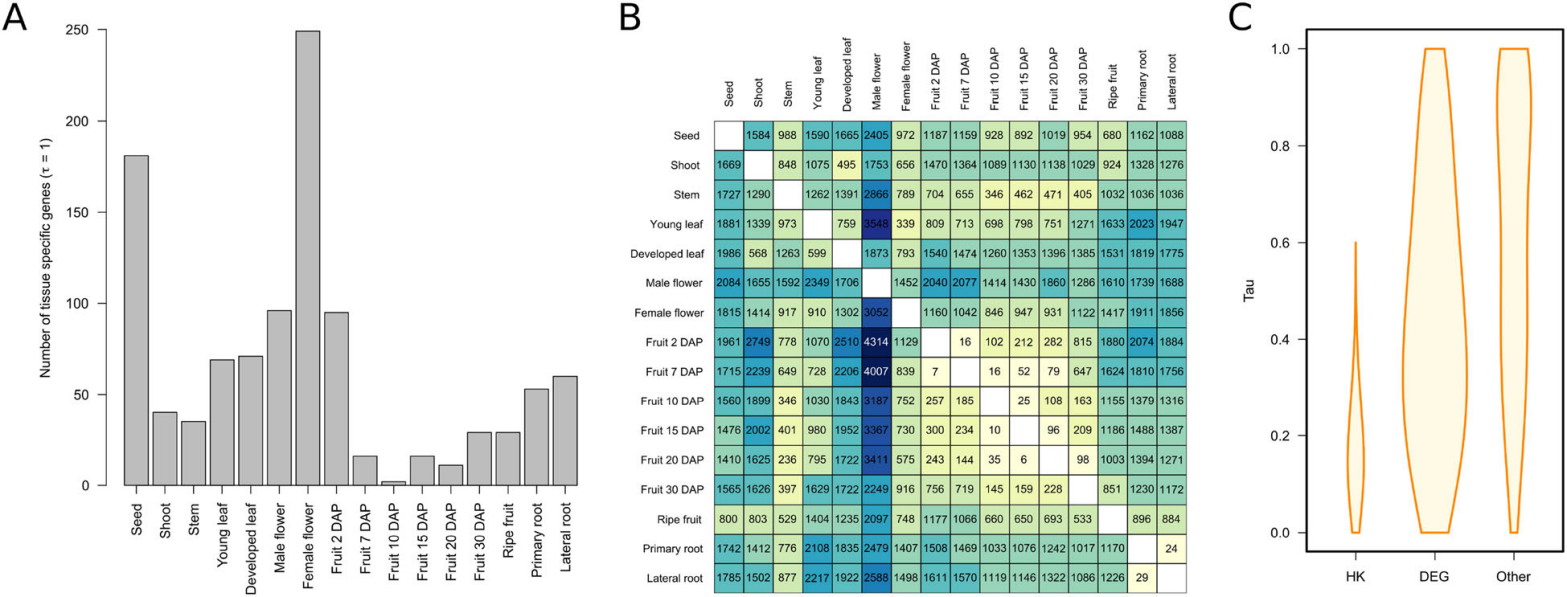
**a** Distribution of the number of tissue-specific genes among tissues. **b** Heatmap of the number of upregulated genes [log2 (fold change) ≥ 2 and adjusted *P* ≤ 0.01] between pairs of tissues when comparing the tissues from the rows with those from the columns. Color scale varies from yellow (lowest number of genes) to dark blue (highest number of genes). **c** Distribution of gene-tissue specificity measured as τ among putative housekeeping genes (HK), genes found to be differentially expressed between pairs of tissues (DEG), and the rest of the genes

Some genes were solely or mainly expressed in specific tissues, so they were thought to be responsible for specific functions of the corresponding organs. The tissues with the greatest number of specific genes were seeds (178), female flowers (157), male flowers (120), and 2DAP fruits (77), whilst intermediate-age fruits from 7DAP to 20DAP had the fewest (Fig. 3a**;** Table S3). Fruits at 10DAP had only three specific genes, orthologues of *GMP* synthase, ubiquitin C, and interleukin-1 receptor-associated kinase, indicating that although the fruit differed in morphology, its transcriptome cannot be easily distinguished from the other fruit tissues.

GO terms and KEGG pathway analysis were used to classify the functions of the specific genes for each tissue (Table S3). On the basis of sequence homology, the two categories frequently represented within the different tissues were carbohydrate metabolic process and cell redox homeostasis from BP classification, as well as polygalacturonase (PG) activity, protein disulfide oxidoreductase activity, and terpene synthase activity from MF classification. In the same context, important over-represented pathways of tissue-specific genes included plant hormone signal transduction and pentose and glucuronate interconversions.

Seeds had 178 tissue-specific genes, related to cell wall organization, carbohydrate metabolic process, and lipid transport (Fig. 3a**;** Table S3), with genes exhibiting PG activity being over-represented. Activity of PGs, which belong to one of the largest hydrolase families, are associated with a broad number of developmental changes, including seed germination and embryo development. In fact, PGs were found in the endosperm of tomato (*Solanum lycopersicum* L.) seeds, being most activated during seed germination [20]. Seven PG-like genes were identified as seed-specific (Table S3). Furthermore, several ethylene-responsive TFs were exclusively expressed in the seeds, including RAP2–3 (BGI_novel_G001750 and BGI_novel_G001751), TINY (Cp4.1LG02g14570), and CRF2-like (Cp4.1LG05g06240). Seed germination and dormancy have been previously correlated with ethylene production, by regulating abscisic acid metabolism and other hormonal signaling pathways [21].

Shoots, young leaves, and developed leaves had 42, 52, and 65 tissue-specific genes, respectively (Fig. 3a), mainly assigned to cell redox homeostasis and plant hormone signal transduction pathway (Table S3). This is indicative of the substantial differences in the transcriptome of the young as compared with the fully developed leaves. Several TFs were solely expressed in young leaves, including the ethylene-responsive TFs ERF096-like (BGI_novel_G000006) and CRF2-LIKE (Cp4.1LG05g03010), and other TFs, such as MUTE (Cp4.1LG08g04260), and SPEECHLESS (Cp4.1LG09g00440), known to be involved in stomata development [22], or in developed leaves, including TCP18-like (Cp4.1LG01g13580) and RADIALIS-like (Cp4.1LG07g04080 and Cp4.1LG15g05490), likely involved in leaf senescence [23], depicting the different biological processes that are boosted or repressed during leaf development.

The female flower had 157 tissue-specific genes (Fig. 3a), mostly associated with cell wall and metabolic processes, including pectin catabolic process, cell wall modification and carbohydrate transport (Table S3). Others included the pollen allergen Ole e 6-like genes, which may be involved in recognition between pollen-stigma and pollen tube-style cells, as well as pollen tube cell-wall proteins known as leucine-rich repeat extensins (such as Cp4.1LG17g03640) that are upregulated during pollen germination and pollen tube growth [24]. Another interesting TF with specific expression in female flowers was the novel gene BGI_novel_G001938, annotated as the VIN3-like protein 2, likely involved in both the vernalization and photoperiod pathways promoting flowering under specific photoperiod conditions [25].

The male flower had 120 tissue-specific genes **(**Fig. 3a). Similarly to female flowers, the carbohydrate metabolic process was activated. Male flowers specifically expressed some genes known to be involved in flowering, such as an EPIDERMAL PATTERNING FACTOR-like protein 6 (Cp4.1LG20g07670) that might act primarily as positive regulator of inflorescence growth [26] (Table S3). Ethylene is the most important factor regulating sex expression, controlling the transition from male to female flowering, as well as the ratio of female to male flowers, and sex determination of individual floral buds [27]. Genes of the *ERF* family, such as the male flower-specific ethylene-responsive transcription factor 2-like (Cp4.1LG13g02430), may be involved in ethylene signaling associated with male flowering in *Cucurbita*.

The young fruits, at 2DAP, had 77 tissue-specific genes (Fig. 3a), likely associated with the unique processes that convert the ovary of the female flower into a fruit. The most over-represented biological processes in young fruits (Table S3) were associated with metabolic, developmental, and biosynthetic processes, including polyprenol biosynthetic process, and sesquiterpene biosynthetic process. Specific genes of 2DAP fruit included several enzymes involved in the synthesis of terpenes, monoterpenes and sesquiterpenes, compounds known to be involved in cucurbit-fruit aroma [28]. The specific expression of the ethylene responsive factors (ERFs) BGI_novel_G002208, Cp4.1LG11g00790, and Cp4.1LG11g00790, as well as the ethylene biosynthetic enzyme 1-aminocyclopropane-1-carboxylate synthase (ACS; Cp4.1LG18g03790) was evident. The later one corresponds to the *C. pepo* gene *CpACS27A*, orthologous to the *Cucumis melo* gene *CmACS7* (MELO3C015444), responsible for the andromonoecious phenotype and fruit length [29, 30]. The expression of *CmACS7* during flower differentiation inhibits the development of stamen primordia and leads to unisexual female flowers via an unspecified non-cell-autonomous mechanism [31]. *CpACS27A* has been previously reported to be expressed in squash female flowers and it also has a role in the control of andromonoecy-associated traits, such as the delayed maturation of corolla and stigma as well as fruit parthenocarpic development [32].

Intermediate and later stages of fruit development had a much lower number of tissue-specific genes, ranging from only 3 (at 10DAP) to 23 (at 30DAP and ripe fruit) (Fig. 3a; Table S3). In ripe fruit, the GO terms oxylipin biosynthetic process and glucose transmembrane transporter activity were over-represented. Also, ripe fruits specifically expressed the transcription repressor OFP8-like (Cp4.1LG11g01890), a member of the Ovate Family Proteins, which are involved in fruit morphology and other plant growth and developmental processes [33].

The primary root had 56 tissue-specific genes (Fig. 3a; Table S3). The GO-term enrichment analysis of primary root-specific genes showed an over-representation of calmodulin binding molecular function, with several novel calmodulin-binding proteins specifically expressed. These proteins are involved in many plant processes including root elongation and gravitropic response, and are known to be differentially expressed in different tissues in a spatio-temporal manner [34]. The KEGG pathways plant-pathogen interaction and the MAPK (mitogen-activated protein kinase) signaling were both activated in the primary root.

The lateral root had 40 tissue-specific genes (Fig. 3a; Table S3). The most overexpressed GO classification was metal ion transport. Several copper and nitrate transporters were among the lateral root-specific genes, including Cp4.1LG00g07400 (copper-transporting ATPase) and the NRT1/ PTR FAMILY 6.3-like (Cp4.1LG13g02260). This latter gene is the orthologue of AT1G12110.1, a dual-affinity nitrate transporter expressed in lateral roots, involved in nitrate signaling, stimulating lateral root growth [35]. Also, the lateral roots specifically expressed the biosynthetic enzyme *ACS* (Cp4.1LG19g10460), probably involved in stress sensoring and signaling.

### Differentially expressed genes between tissues

Apart from the genes expressed in specific tissues, there were also differentially expressed genes (DEGs) between the various tissues (Fig. 3b) depicting differential expression between specific tissue pairs. The DEGs showed a wide range of τ, ranging from completely tissue-specific (τ = 1.00) to widely expressed, with a median of 0.42 (Fig. 3c). Tissue-pairs with the highest number of upregulated genes were the first stages of fruit development (2DAP and 7DAP) and young leaf paired with male flowers (more than 4000) (Fig. 3b). In fact, the male flower was the tissue with more DEGs when paired with the remaining tissues, even more than the seed and the shoot, whilst the leaf stem was the tissue with the least DEGs. The pairs of tissues that had the fewest DEGs were fruits at 2DAP and 7DAP, as well as fruits at 10DAP and 15DAP, indicative of similar transcriptome profiles. Many of these genes are likely involved in the biochemical changes that occur during the manifold biological processes (Table S4).

Male-flower tissue differed from female-flower tissue in 3418 genes upregulated in female compared to male flowers, and 2517 genes upregulated in male compared to female (Fig. 3b). Main GO terms enriched in genes upregulated in the female compared to male flowers were related to translation, ribosome biogenesis, ribosomal large and small subunit assembly, auxin-activated signaling pathway, cell wall modification, and pectin catabolic process (Table S5). Many pectinesterases and PGs, as well as other cell-wall related enzymes and sugar transporters, were upregulated in female flowers. By contrast, GO terms overexpressed in genes upregulated in male compared to female flowers were associated with different general BPs, such as photosynthesis, tricarboxylic acid cycle and autophagy, or the specific process of pollination and anther development.

By comparing DEGs in flowers and fruits (2, 7, 10, 15, 20, 30 DAP and 40DAP ripe fruit) (Table S6), GO enrichment analysis showed that flowering-specific BPs, such as anther development and pollination were activated more in flowers than fruits. However, other metabolic pathways such as those related to carbohydrate metabolic process and cell-wall related process including cell wall organization, pectin catabolic process, and cell wall modification, were also overrepresented. Photosynthesis and transcription terms were overrepresented in fruits. An interesting note is that the principal cellular compartment of DEGs upregulated in flowers was the extracellular region, whilst in fruits, it was the chloroplast thylakoid membrane.

Differential gene expression also occurred over the course of fruit development (Fig. 3b). For example, 36 DEGs differentiated fruits at 2DAP and 7DAP, with 25 of them up-regulated in 2DAP and 11up-regulated in 7DAP (Tables S4). There were two main expression changes during fruit development: the first at the beginning, from female flower to fruit at 2DAP (with 1484 genes upregulated in female flowers and 1269 in 2DAP fruits), and the second at the end, from fruits at 30DAP to ripe fruits-40DAP (with 1054 upregulated in 30DAP and 783 in ripe-40DAP fruits). Fewer changes were observed among the intermediate fruit stages but, even so, there were two key points, changes from fruits at 7DAP to fruits at 10DAP (27 and 250 DEGs, respectively) and from fruits at 20DAP to fruits at 30DAP (141 and 321, respectively).

The changes that occur during the transition from female flower to fruit at 2DAP were intriguing (Tables S4-S5). The main GO term over-expressed in upregulated genes in 2DAP fruit as compared with female flowers, but also in intermediate fruit stages (to 20DAP) as compared with ripe fruit, was microtubule-based movement, as many kinesin proteins were upregulated in these stages. These are microtubule-based motors responsible for modulating cell division and enlargement, and are known to be involved in cell division and expansion in early fruit development [36]. By contrast, the dominant GO terms over-expressed in upregulated genes in ripe fruit as compared with the rest of the fruit stages were translation and photosynthesis, and light harvesting. The main over-represented KEGG pathways in ripe fruits as compared with the other fruit-development stages were plant-pathogen interaction, plant hormone signal transduction, and phenylpropanoid biosynthesis.

Apart from the specific genes found in primary and lateral roots (described above; Fig. 2b), these two tissues only differ in 40 and 33 genes upregulated in primary and lateral, respectively (Table S4). The root phototropism 2-like protein (Cp4.1LG02g11200), which is involved in root phototropism, as well as hypocotyl phototropism under high-rate light in *Arabidopsis* [37], was clearly up regulated in lateral roots.

The RNA-Seq-based profiles of a selection of nine key genes, differentially expressed between group of tissues, was further compared with quantitative Real-Time (qRT)-PCR based gene expression values between different groups i.e. male and female flower, young and developed leaf, 2DAP fruit and 40 DAP fruit, as well as primary and lateral root (Fig. S1). Results indicated a strong positive correlation of the expression profiles between the two methods. The primary functions of the nine verified genes were related to flowering, ethylene biosynthesis and perception, fruit morphology, and ascorbate biosynthesis. Expression data were normalized against a common reference gene, elongation factor-1A (Cp4.1LG12g00880) [38, 39], validated in zucchini, as well as two putative HKG from Table S2, that exerted stable expression across our data set based on RNA-Seq results (Table S7). These data confirmed the reliability of the RNA-Seq results obtained in this study, and further suggested new valuable HKG for data normalization in summer squash that should be further validated in other genotypes or in plants subjected to stress too.

#### Identification of co-expression groups by weighted gene co-expression network analysis (WGCNA)

**Fig. 4:**
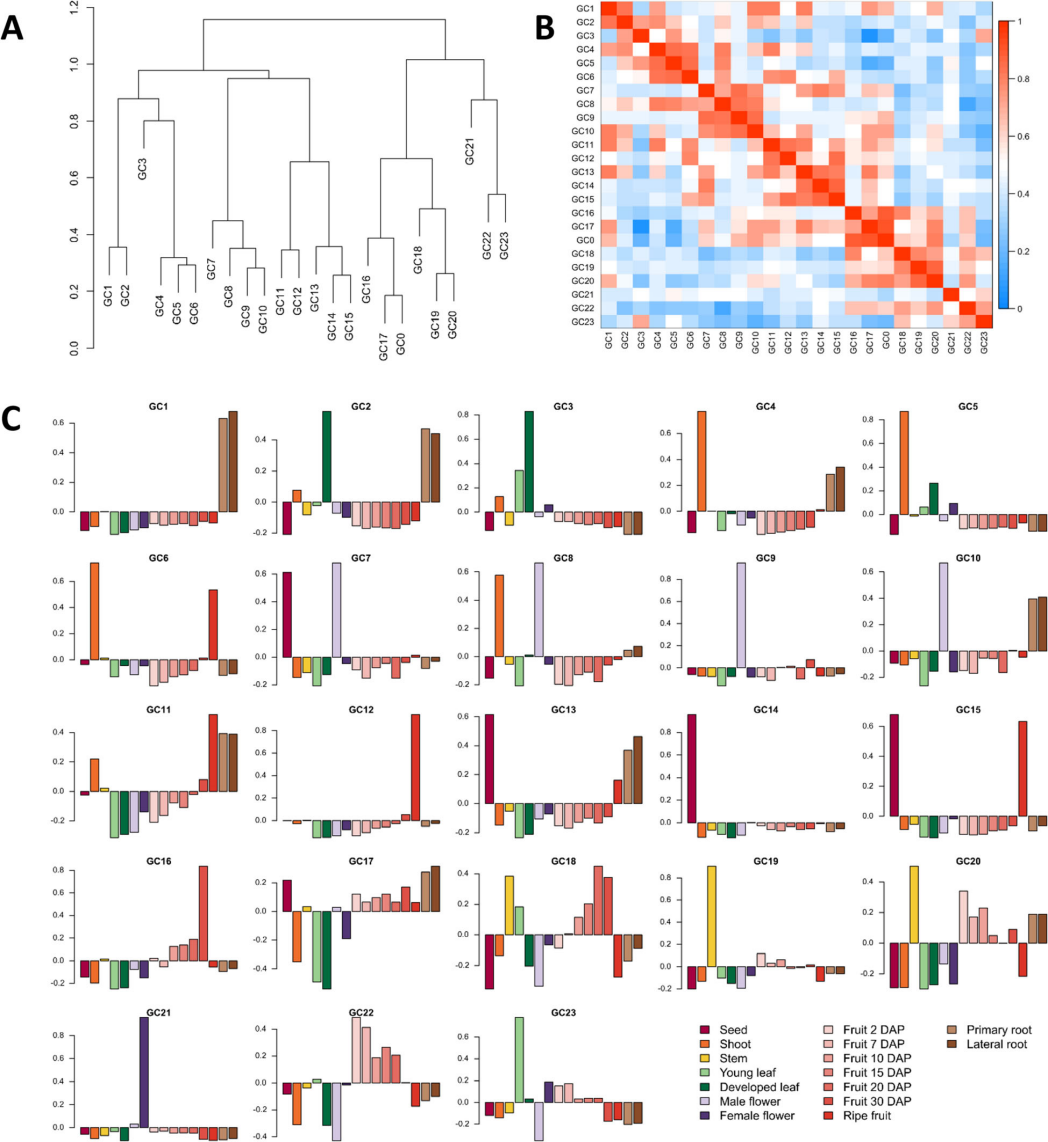
**a** Hierarchical clustering of eigengenes of the co-expressed gene clusters found by weighted gene coexpression network analysis. **b** Heatmap of the relationship among gene clusters (measured as the eigenge adjacency). **c** Gene expression patterns across tissues for each gene cluster. Eigengene values are represented across tissues

Following WGCNA analysis, 25,413 genes were classified into 24 gene clusters (GCs) (Fig. 4). The number of genes in these GCs ranged from 305 (GC24) to 4523 (GC23). Some GCs exhibited clear gene expression tissue specificity, such as GC1 (root), GC3 (leaf), GC4 (shoot), GC5 (shoot), GC9 (male flower), GC12 (ripe fruit), GC14 (seed), GC16 (30DAP fruit), CG19 (leaf stem) and GC21 (female flower) (Fig. 4c). The genes included in each CG, as well as GO and KEGG terms enrichments are included in Tables S8 and S9, respectively. Some pairs of tissues exhibited specific expression of GC, such as roots and developed leaves (GC2), male flower (GC10), or ripe fruit (GC11), male flower and seed (GC7) or shoot (GC8), and ripe fruit and seeds (GC15) or shoot (GC6). It is likely that genes classified into such ‘tissue-overlapping’ GCs are involved in common molecular processes in different tissues. Furthermore, GC22, GC18 and GC20 exhibited fruit-specific or preferential gene expression, as their levels were found to be high from 2DAP to 30DAP, implicating them in fruit development and ripening. Similar results were obtained by Yano et al. [13], in an RNA-Seq based transcriptome analysis on 30 different tissues of the ‘Harukei-3’ melon that classified 17,597 genes into 45 GCs.

GC1 was enriched for root-specific genes, and GO term enrichment confirmed GC1 was enriched in genes associated with the ‘nucleus’ cellular component (Table S9). Reproductive-specific genes were enriched in GC21 corresponding to GO enrichment terms of “pollination”. Four groups (GC7, GC13, GC14 and GC15) were enriched for seed-specific genes with GO term enrichment classes of ‘integral component of membrane’ and ‘nutrient reservoir activity’. GC3 was enriched for leaf-specific genes and GO enrichment identified ‘photosystem II’ and ‘photosynthesis’. Finally, GC22 was jointly enriched for fruit development-specific genes corresponding to GO enrichment terms of ‘fruit morphogenesis’ and ‘microtubule motor activity’. The KEGG pathways of phenylpropanoid biosynthesis, and starch and sucrose metabolism were significantly enhanced in GC16 and GC12, respectively, corresponding to fruits at 30DAP and 40DAP.

#### Flowering-related genes

Several flowering-related genes were similarly expressed at the majority of tissues, as well as fruit developmental stages (Fig. S2). These genes included orthologues of APETALA-2, EARLY FLOWERING 3 and 4, Flowering Control protein (FCA), FLOWERING LOCUS T protein, and Flowering time control FPA-like protein among others. An interesting note is that between male and female flowers, genes related to the FLOWERING LOCUS T (Cp4.1LG14g06110, Cp4.1LG18g02360), and EARLY FLOWERING 3 (the two paralogous genes Cp4.1LG19g10710 and Cp4.1LG10g02240), were significantly upregulated in male flowers compared to female flowers (Table S4). The gene Cp4.1LG18g02360 was much less and more specifically expressed than its paralogue Cp4.1LG00g01430. By contrast, orthologues of the flowering promoting factor 1 (Cp4.1LG16g06230), of the EARLY FLOWERING 3 (Cp4.1LG05g11750), and of the Ultrapetala (Cp4.1LG05g14680) proteins were mostly upregulated in female flowers compared to male flowers. Male flowers and the late stages of fruit development overexpressed the Unusual floral organs (Cp4.1LG03g13830 and Cp4.1LG03g13810) known to be involved in floral meristem determination. Some of the genes studied as flowering related can be also more associated to other processes. For example, the protein MOTHER of FT and TFL1 protein (Cp4.1LG09g11210), known to be a key regulator of embryo development and seed germination [40], which is consistent with its upregulation in seeds and ripe fruit (Fig. S2), and Ultrapetala (Cp4.1LG15g07920) significantly upregulated in roots. Other flowering promoting factors, including Cp4.1LG03g11390 and its paralogues Cp4.1LG17g00630, Cp4.1LG08g04810, and Cp4.1LG12g02680, were highly and/or almost specifically expressed in roots, whereas Cp4.1LG16g06230 was expressed in female flowers, all fruit tissues and seeds, but not in roots. These different expression profiles can help to identify those genes involved in similar functions in different tissues. In this regard, the roots may not only be involved in root functions, but also interact with other important above-ground plant processes, for example flowering, either by being on the route of systemic signals or by participating actively in its regulation.

The AGAMOUS-like MADS-box transcription factor Cp4.1LG02g10680, the orthologue of AT4G09960, which is involved in fertilization and seed development [41], controlling fruit size by regulating cytokinin levels, was consistently upregulated in seeds and increasingly upregulated as fruit ripens (Fig. S3). Transcription factors involved in the genetic control of flower development, such as the PISTILLATA genes (Cp4.1LG02g03120 and Cp4.1LG06g06100), were upregulated in flowers and less expressed in the three earliest fruit stages.

#### Ethylene-related genes

As for the ethylene metabolic pathway, a significant upregulation of the 1-aminocyclopropane-1-carboxylate oxidase (*ACO*; Cp4.1LG04g02610) was observed at intermediate stages of fruit ripening, as well as male flowers (Fig. S4a), compared with vegetative and root tissues. Its paralogue (Cp4.1LG05g15190), also expressed in all tissues except seeds, clearly displayed higher expression in vegetative tissues. On the other hand, *CpACO2A* (Cp4.1LG19g08030), the orthologue of *CsACO2* and *CmACO3*, involved in the promotion of carpel development, and its paralogue *CpACO2B* (Cp4.1LG10g09730), showed lower expression levels and were barely detected in seeds, male flowers and most fruit tissues, although maintaining high expression levels in female flowers. By contrast, Cp4.1LG07g10650 was the only *ACO* specifically expressed in root tissues.

The expression of *CpACS27A* (Cp4.1LG18g03790), responsible for the andromonoecious phenotype [29], was detected only in the early fruit stage at low levels (2DAP), whilst its paralogue, *CpACS27B* (Cp4.1LG04g10620) was highly expressed in all tissues (Fig. S4b). *CpACS27A* has been reported to be specifically expressed in female flowers, being responsible for unisexual female flowers [31]. In our study, the orthologue of *CmACS11* (Cp4.1LG11g01010), the androecious gene involved in the promotion of carpel development [29] showed a progressive upregulation as the fruit ripens, whereas another *C. pepo* orthologue of *ACS* (Cp4.1LG00g10840) was progressively downregulated from the early stage of fruit set. Other *ACS* genes show low expression levels in all or specific tissues (seeds, stems, different fruit developmental stage or roots).

Apart from *ACS7* and *ACS11*, a third master gene controlling sex determination in cucurbits is the gynoecious *locus CmWIP1*, previously reported to be involved in the occurrence of gynoecy in melon [42]. The *CmWIP1* transcription factor has two orthologues in *C. pepo*, *CpWIP1A* (Cp4.1LG05g05020), expressed in all tissues, but presumably in female flowers, and *CpWIP1B* (Cp4.1LG16g08860), which has a very low expression mainly detected in developing fruit (Fig. S4c).

Besides the ethylene biosynthesis enzymes, ethylene receptors that act in ethylene signaling are involved in sex determination and other plants processes [32], serving as downstream components of ethylene signaling, mediating ethylene-dependent gene transcription. Some of these factors show enhanced expression at the onset of ripening while others display a ripening-associated decrease in expression (Fig. S4c), suggesting that different *ERF*s may have contrasting roles in flower development and fruit ripening. For instance, the male flower-specific ethylene-responsive transcription factor 2-like (Cp4.1LG13g02430), may be involved in ethylene signaling associated with male flowering. On the other hand, *CpETR1A* (Cp4.1LG07g07800), *CpETR1B* (Cp4.1LG11g06650), *CpETR2A* (Cp4.1LG03g10030) and *CpETR2B* (Cp4.1LG08g03550) were expressed in all tissues, with similar expression in male and female flowers, except for *CpETR2A*, which is more expressed in male flowers and with a gradual increase in fruits during ripening (Fig. S4c). This behavior is consistent with their reported role in the development of floral organs in pistillate flowers, including ovaries and fruits [32]. On the other hand, *ERS1A* (Cp4.1LG12g05940) seemed to be functional and expressed in all tissues, whereas *ERS1B* was only barely detected in female flowers and young fruits. Other *EFR*s (Cp4.1LG09g08200 and Cp4.1LG19g07200) showed decreasing expression as fruit ripens.

Several genes of the Ethylene-Insensitive 3 (*EIN3*) family, involved in signaling, also showed clear differences in their levels of expression (Fig. S4c). Among them, the most expressed was Cp4.1LG04g11790, one of the two *EIN3* genes that underlies a QTL related with early flowering [9, 10]. These findings are indicative of the paramount role of ethylene in sex expression and fruit ripening [27].

#### Fruit morphology-related genes

The *C. pepo* genome includes more than 20 genes annotated as Ovate Family Proteins (Fig. S5a), controlling multiple aspects of plant growth and development [33]. A gene of this family (Cp4.1LG03g03420) has been previously reported to underly a major QTL involved in fruit shape 10]. Consistent with its role in fruit morphology, this gene was found specifically expressed in the early fruit stages, as well as in roots. The paralogue of this gene, Cp4.1LG10g07570, was highly expressed in roots and less in the remaining tissues. Consistently with the wide role of Ovate genes in different developmental processes, Cp4.1LG12g08240 was overexpressed in roots, whereas Cp4.1LG01g025030 in seeds.

The DELLA protein GAIP-B orthologue (Cp4.1LG04g09740) was overexpressed in female flowers compared to male flowers (Fig. S5b), being constantly expressed though the ripening process and significantly overexpressed at ripe fruits. These proteins have been proposed to serve as repressors of the gibberellin (GA) signaling pathway, probably by repressing the transcription of GA-inducible genes. On the other hand, GA20-oxidase 1 genes (Cp4.1LG09g01290 and Cp4.1LG09g01310) were specifically expressed in the seeds and ripe fruits, while Cp4.1LG14g03420, and Cp4.1LG20g05150 overexpressed in seeds and intermediate stages of fruit ripening, indicating that gene copies may have distinguished functions in individual tissue developmental stages. Previously, GA20-oxidases have been demonstrated to regulate various aspects of vegetative growth, including floral and anther development in *Arabidopsis* [43] but their role in cucurbits is less clear.

Fruit tissues at early development overexpressed several *WUSCHEL* TFs (Cp4.1LG19g06930, Cp4.1LG09g11150 Cp4.1LG10g05160 and Cp4.1LG16g05720) (Fig. S5c), which are known to contribute to enlarging fruit size by altering meristem activity. Other copies, such as Cp4.1LG00g014720 and Cp4.1LG11g01360, were upregulated in roots as compared to the other fruit or vegetative tissues, while Cp4.1LG07g05080 was overexpressed in lateral roots as opposed to primary roots, which is in accordance with their proposed role in regulating the formation and regeneration of lateral roots in cucumber [44]. Genes of the plant-specific YABBY transcription factor family showed higher expression in female flowers compared to male flowers (Fig. S5d), suggesting their putative role in modulating early reproductive organ development. Further, several orthologues were overexpressed in seeds compared to other fruit or vegetative tissues, indicating their potential involvement in seed development, as previously reported in grapevine [45].

#### Fruit quality related genes

**Fig. 5:**
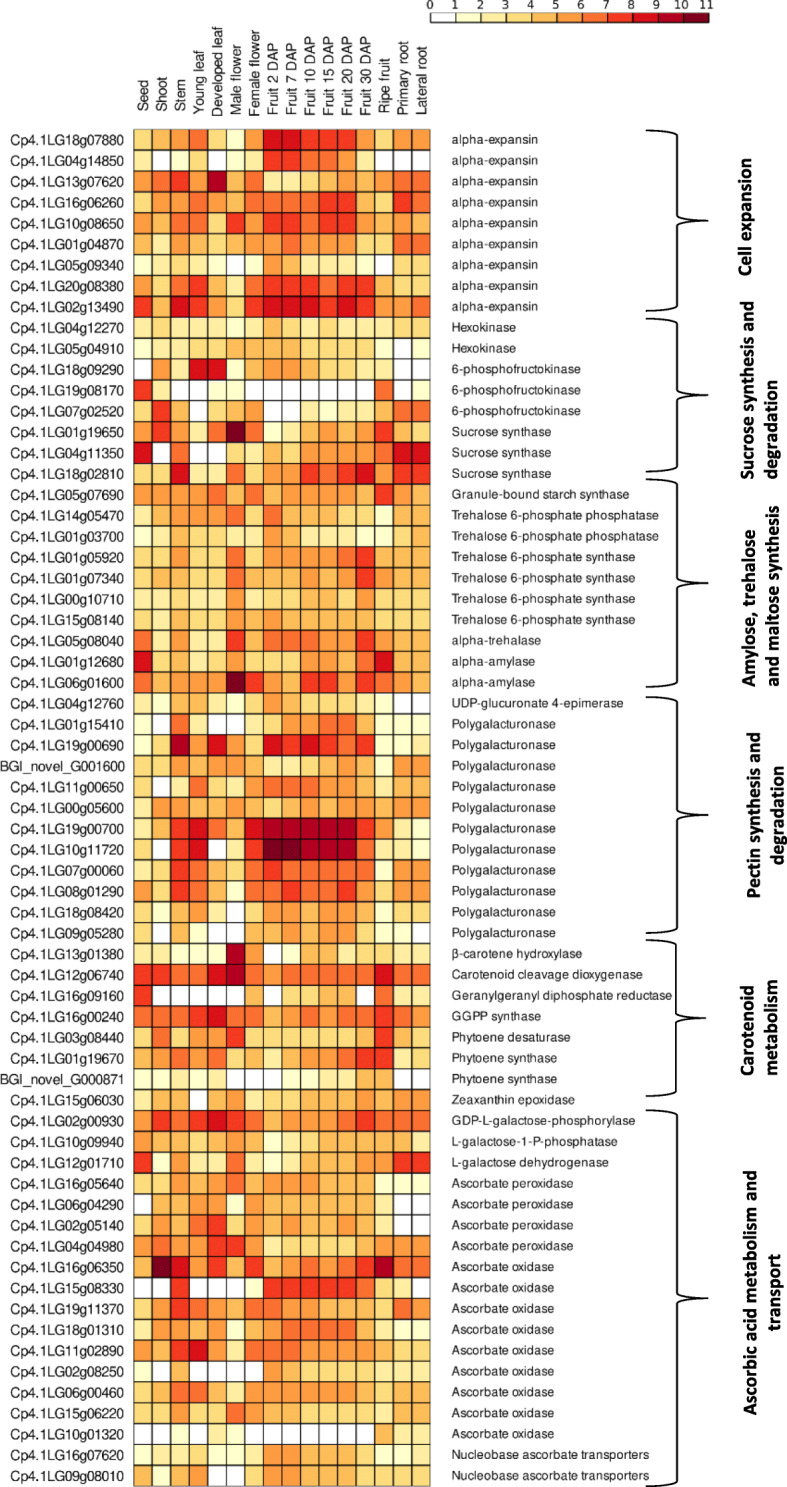
Expression profiles of key genes related to fruit quality, and in particular cell expansion, sucrose synthesis and degradation, amylose, trehalose and maltose synthesis, pectin synthesis and degradation, carotenoid metabolism, as well as ascorbic acid metabolism and transport, across the different plant tissues and fruit developmental stages. Gene annotation information of *C. pepo* genome v.4.1 (right labels) is also provided. The colour scale represents log2 of normalized FPKMs

Expansins (EXPA) are non-hydrolytic proteins that are able to trigger a pH-dependent re-modelling and loosening of the cell wall, enabling cell expansion, organ abscission, and fruit softening [46]. A significant downregulation of several putative alpha-*EXPA* was evident especially after 30DAP (Fig. 5), which coincided with the induction of ethylene biosynthetic genes (Fig. S4). These results reinforce the positive effect of ethylene on cell expansion by regulating genes of the EXPANSIN family, in spite of the fact that a direct regulatory mode of action between *EXPA* and ethylene is still missing. Two orthologues of alpha-EXPA (Cp4.1LG18g0788 and Cp4.1LG02g13490) seemed to be fruit-specific, as they were highly expressed throughout the ripening process, and significantly down-regulated at ripe fruits.

Many structural genes involved in carbohydrate metabolism, in particular sucrose and starch metabolism, have been identified as DEGs within our dataset (Fig. 5). Specifically, genes involved in sucrose degradation such as hexokinases (Cp4.1LG04g12270, Cp4.1LG05g04910), in trehalose synthesis such as trehalose phosphate phosphatase (Cp4.1LG14g05470, Cp4.1LG01g03700) and alpha-trehalase (Cp4.1LG05g08040), in pectin synthesis and degradation including UDP-glucuronate 4-epimerase (Cp4.1LG04g12760) or *PG*s (such as Cp4.1LG19g00690, Cp4.1LG19g00700 and Cp4.1LG10g11720), that were abundant early in fruit development, were mostly reduced at ripe fruits (40DAP). Against this backdrop, other genes related to sucrose degradation, such as phosphofruktokinases (Cp4.1LG19g08170, Cp4.1LG07g02520), to sucrose synthesis such as sucrose synthases (Cp4.1LG01g19650, Cp4.1LG04g11350, Cp4.1LG18g02810), to trehalose synthesis, such as trehalose phosphate synthases (Cp4.1LG01g05920, Cp4.1LG01g07340, Cp4.1LG00g10710), to maltose synthesis, such as alpha-amylase (Cp4.1LG01g12680), as well as to pectin degradation, including less abundant *PG*s, such as Cp4.1LG01g15410, BGI_novel_G001600, and Cp4.1LG00g05600, were induced over the course of fruit ripening. Most of these results are similar to those reported for *C. maxima* transcriptomic profiling during ripening [14]. Granule-bound starch synthase, a gene responsible for amylose synthesis, which directly affects both the taste and texture of fruits and their postharvest storage was also significantly over-expressed in ripe fruits (Cp4.1LG05g07690). This is in accordance with previous studies demonstrating that its expression and activity is induced in ripening fruits such as banana (*Musa spp.*) [47]. Trehalose-6-Phosphate is an intermediate product of trehalose biosynthesis, produced in a reaction catalyzed by trehalose-6-phosphate synthase, and is further metabolized to trehalose by trehalose-6-phosphate phosphatase, which, in turn, is hydrolyzed into glucose by alpha-trehalase. Results reported herein indicated that although trehalose-6-phosphate synthase expression was mostly enhanced during fruit ripening, both trehalose-6-phosphate phosphatase and alpha-trehalase transcript levels dropped early in ripening or at 40DAP, respectively (Fig. 5), probably leading to a higher accumulation of trehalose-6-Phosphate. This intermediate precursor of trehalose has a dual function in sucrose sensing and regulation, exerting a pivotal role in both plant development and abiotic stress tolerance [48]. Although the majority of these genes significantly contribute to fruit softening and taste, an enhanced expression of several of them was also identified in other vegetative part of *C. pepo*, such as roots, leaves and flowers.

Several structural genes from the carotenoid metabolic pathways were upregulated, especially at the later stages of ripening (Fig. 5), probably resulting in enhanced accumulation of carotenoids in ripe fruits. In particular, these genes included β-carotene hydroxylase (Cp4.1LG13g01380), carotenoid cleavage dioxygenase (Cp4.1LG12g06740), geranylgeranyl diphosphate reductase (Cp4.1LG16g09160) and synthase (Cp4.1LG16g00240), phytoene desaturase (Cp4.1LG03g08440), phytoene synthase (Cp4.1LG01g019670 and BGI_novel_G000871), and zeaxanthin epoxidase (Cp4.1LG15g06030). The expression of β-carotene hydroxylase and zeaxanthin epoxidase determines the accumulation of zeaxanthin and violaxanthin, respectively [14], whilst carotenoid cleavage dioxygenases are critical enzymes catalyzing the generation of apocarotenoids, which in turn orchestrate carotenoid biosynthetic flux, exerting major functions in stress signaling and aroma development.

The ascorbic acid (AsA) biosynthetic pathway driven through _L_-galactose was strongly induced from 20DAP onwards, as indicated by the upregulation of APE), and to a lesser extent of _L_-galactose-1-P-phosphatase (Cp4.1LG10g09940), and _L_-galactose dehydrogenase (Cp4.1LG12g01710) (Fig. 5). In ripe apples [49] and tomatoes [50], GDP-_L_-galactose-phosphorylase has been identified as the rate-limiting step in AsA accumulation. In contrast, several orthologues of ascorbate peroxidase and the apoplastic ascorbate oxidase from the ascorbate recycling and degradation routes, respectively, were significantly repressed at the later stages of ripening, with a few exceptions. The members of ascorbate oxidase of the *Cucurbitaceae* family are the most abundant sources of this enzyme within plant kingdom. Although ascorbate oxidase has been mostly correlated with cell expansion early in fruit ripening, recent results in melon demonstrated an ascorbate oxidase role at later developmental stages, leading to modifications in fruit size, but also an alternative route for AsA enhancement in ripening fruit [51]. Additionally, the nucleobase ascorbate transporters (Cp4.1LG16g07620 and Cp4.1LG09g08010) were upregulated in young developing fruits and leaves, as well as stems, supporting their putative role in long-distance transport of AsA [49].

## Conclusions

In the present study, we developed the first Gene Expression Atlas (CupeGEA) for a *C. pepo* subsp. *pepo* summer squash cultivar based on 16 vegetative and fruit tissues during development and ripening. In total, 27,868 genes and 2352 novel transcripts were annotated from these 16 tissues, with over 18,000 genes common to all tissue groups. A broad number of DEGs were identified between the various tissues, the male flower being the tissue with the most differentially expressed genes in pair-wise comparisons with the remaining tissues, and the leaf stem the least. The largest expression change during fruit development was early on, from female flower to fruit two days after pollination. The WGCNA performed on the global gene expression dataset assigned 25,413 genes to 24 coexpression groups, and identified group of genes with strong tissue specificity. This gene expression atlas of squash is expected not only to provide a global view of gene expression patterns in all major tissues and fruit developmental stages in *C. pepo* but to also serve as a valuable resource for functional genomics accelerating gene discovery in the *Cucurbitaceae*.

## Methods

### Plant growth and sample collection

Seeds of the Greek summer squash ‘Kompokolokytho’ are maintained in the seed collection of varieties at the Institute of Plant Breeding and Genetic Resources of the Hellenic Agricultural Organization (HAO) DIMITRA. Plants of ‘Kompokolokytho’ have bush growth habit, some basal branching, dark stems, and petioles that are more sharply spiculate than most modern cultivars (Fig. 1). Its staminate flowers are about average in size but the petals of its pistillate flowers are quite large as compared with other summer squash cultivars. The young edible fruits, from 2 to 5 days after pollination, are light-medium green, becoming dark green by 15 DAP, then turning orange upon ripening, 40 DAP. The initial fruits produced by plants of ‘Kompokolokytho’ are of vegetable marrow shape, but subsequently produced, acropetal fruits are of cocozelle shape.

Seeds were germinated in soil composed of ‘Na-tera’ (Mitsubishi Chemical Agri Dream) in the dark at 25–28 °C in May, and seedlings were grown under 9 h light (25 °C)/15 h dark (20 °C) conditions until true leaf expansion. Plants were transferred to a soil composed of ‘Coco-bag’ (Toyotane) and grown under greenhouse conditions. In total, 16 tissues were sampled (Fig. 1): primary and lateral roots, shoot (main stem of the plant), stem (of developed leaves), young and developed leaves, male and female flowers, seeds, and fruits of seven developmental stages (2, 7, 10, 15, 20, 30 and 40 DAP, corresponding to ripe fruits). Female flowers were hand pollinated with the pollen from male anthers that were obtained from the same plant (self-pollination), and one or two fruits were retained on each plant. All tissues except for fruits and seeds were sampled when the plants began to flower. Some pistillate flowers were artificially pollinated and tagged with the date of anthesis, the resulting fruits sampled according to the seven fruit developmental stages. For control of diseases and pests, chemicals were applied as necessary.

### RNA isolation and sequencing

For the isolation of total RNA, plant tissues were frozen in liquid nitrogen and stored at − 80 °C until use. Three biological replicates of each of the 16 tissues were maintained, constituted by sampling at least three plants grown at different intervals and located randomly in the glasshouse. RNA was isolated using the Ribospin™ Seed/Fruit RNA isolation kit (GeneAll, Seoul, Korea) according to the manufacturer’s instructions. Equal amounts of RNA samples from the three independent biological replicates (of each tissue) having RNA Integrity Number value of ≥8 were pooled prior to library preparation and subsequent sequencing [16].

The extracted RNA was checked using NanoDrop 2000 (Thermo, CA, USA) and the RNA concentration and integrity were assessed using the RNA Nano 6000 Assay Kit of the Agilent Bioanalyzer 2100 system (Agilent, CA, USA). Oligo (dT) beads were used to isolate poly(A) + mRNA, which was fragmented to 250 bp. Fragmentation of the RNA and reverse transcription of double-strand cDNA was driven by an N6 random primer. The synthesized cDNA was subjected to end-repair and then was 3′ adenylated. Adaptors were ligated to the ends of these 3′ adenylated cDNA fragments. The ligation products were purified and many rounds of PCR amplification were performed to enrich the purified cDNA template using the PCR primer. Each cDNA library was sequenced in a single lane of the BGISEQ-500 system with a paired-end sequencing length of 100 bp at the Beijing Genomics Institute (BGI-Shenzhen, Denmark).

### RNA-seq data analysis

After removing adapter sequences and trimming low-quality reads using SOAPnuke v1.5.2, the high-quality reads were mapped to the pseudomolecule sequences of *C. pepo* Genome v4.1 [5] using HISAT2 v. 2.1.0 [52]. StringTie v1.3.5 [53] was used to reconstruct transcripts, while Cuffcompare (Cufflinks v2.2.1) [54] was used to compare the reconstructed transcripts to the reference annotation. These transcripts were classified as coding transcripts using CPC (Coding Potential Calculator: http://cpc.cbi.pku.edu.cn/). Novel coding transcripts with reference transcripts were merged to get a complete transcript reference, and downstream analyses were performed based on this reference. Clean reads were mapped again to this new reference using Bowtie2, and log2-transformed fragments per kilobase million (FPKM) values were normalized using a trimmed mean of M-values (TMM) using R package edge [55].

### Tissue expression

The FPKM values were used to explore the relationship between tissue expression profiles using a PCA and a hierarchical clustering based on the Pearson Correlation distance of the 1000 most variable genes. Both analyses were performed using the R functions “prcomp” and “heatmap.2” of the package *gplots*. Tissue- and organ-specificity were scored per gene using τ on normalized read counts across all samples using the R package *tispec*. The values of τ range from 0.00 to 1.00, where the higher the value the more likely the gene is specifically expressed at that stage. For this study, genes with τ = 1.00 were considered tissue-specific. Furthermore, the specificity of each gene for each individual tissue was calculated using the tau expression fraction (tef). Detection of putative housekeeping genes was based on their stable and constitutive expression across tissues [56].

DEGs between pairs of tissues were detected using the Audic-Claverie test that assumes a Poisson distribution and implemented in the ACDtool [57]. Probability values (*P*) were corrected for multiple testing using the R function ‘p.adjust’ and the Benjamini-Hochberg (False Discovery Rate, FDR) method. Genes with an absolute log2(fold-change) ≥ 2 and adjusted *P* ≤ 0.01 were considered as over- or under-expressed. Detection of DEGs between groups of tissues were done via a modified *t*-test using the “Linnorm.limma” function of the *Linnorm* R package. Gene ontology (GO) enrichment analysis of DEGs was performed using the R package topGO [58], while KEGG enrichment analysis [59] using *clusterProfiler*.

A weighted gene co-expression network analysis (WGCNA) was also performed to identify clusters of co-expressed genes using the R package *WGCNA* v.1.69 [60]. For each identified GC, a GO and a KEGG enrichment analysis was conducted as above with the genes that were significantly correlated at *P* < 0.001 with the cluster. Expression maps of genes of interest related to flowering, fruit morphology, ethylene metabolism and pathogen defense were drawn for the different tissues. Candidate and putative genes were selected to explore whether differences were observed among tissues or were tissue-specific paralogues or non-functional paralogues.

### Quantitative real-time PCR

Nine genes with significant differences in RNA-Seq-based expression values between selected tissues, i.e. male and female flower, young and developed leaf, 2DAP fruit and 40 DAP fruit, as well as primary and lateral root, were analyzed by qRT-PCR. First-strand complementary DNA (cDNA) synthesis was performed using the same RNA samples (500 ng) as RNA-sequencing, and isolated from three independent biological replicates, consisted of three individual plants each, using the LunaScript® RT SuperMix Kit for cDNA synthesis (NEB), according to the supplier’s protocol. Gene expression profiles were analysed using the Luna® Universal One-Step RT-qPCR Kit (NEB) in the QuantStudio 5 Real-Time PCR system (Applied Biosystems), following manufacturer’s instructions. Taking into account the remarkably different expression profiles between tissues of summer squash, we have tested some putative HKG obtained in this study (Table S2), to eliminate any abundance-related bias. The stability of expression of these HKG was evaluated in our sample set using NormFinder software (http://www.mdl.dk/publicationsnormfinder.htm) [50]. Eventually, expression data were normalized against three reference genes: the putative reference genes *EF-1d* and *G6PDH* (Table S2), as well as *EF-1α*, a previously validated HKG in zucchini [38, 39]. These genes showed a stability value below 0.4. Primers used and amplicon product sizes are listed in Table S7.

## Supplementary Information

**Additional file 1.**

**Additional file 2.**

**Additional file 3.**

**Additional file 4.**

**Additional file 5.**

**Additional file 6.**

**Additional file 7.**

**Additional file 8.**

**Additional file 9.**

**Additional file 10.**

**Additional file 11.**

**Additional file 12.**

**Additional file 13.**

**Additional file 14.**

## Data Availability

All relevant data can be found within the manuscript and its supporting materials. All of the raw reads generated in this study have been deposited in the public database of National Center of Biotechnology under BioProject PRJNA663796.
