# Supplementary figures and images for: A comprehensive RNA-Seq-based gene expression atlas of the summer squash (Cucurbita pepo) provides insights into fruit morphology and ripening mechanisms
Source: BMC Genomics. 2021 May 12;22:341. doi: 10.1186/s12864-021-07683-2 (PMC8114506; doi:10.1186/s12864-021-07683-2)

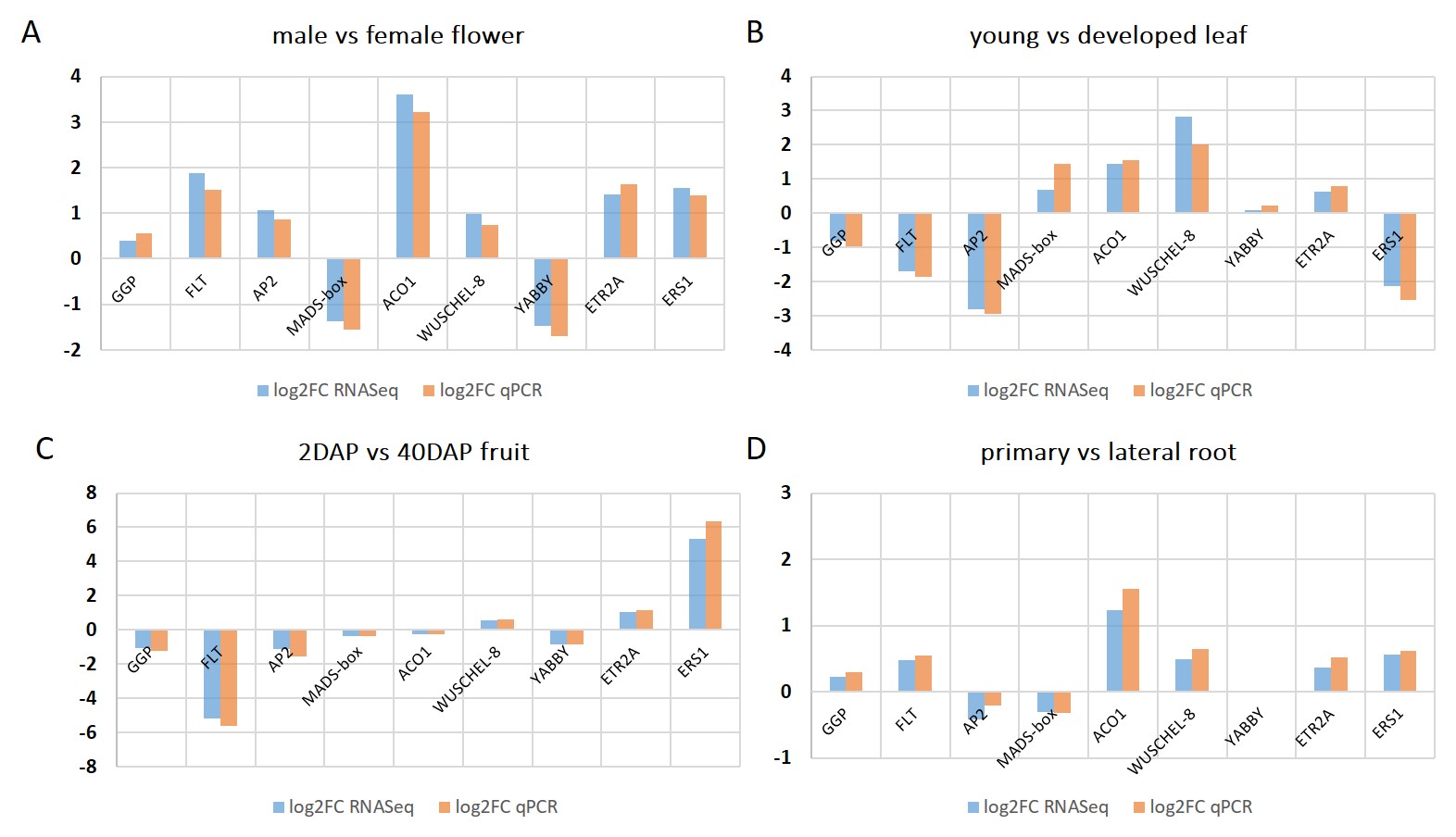

Supplement: Supplementary file 10 — Additional file 10. [file 12864_2021_7683_MOESM10_ESM.jpg]

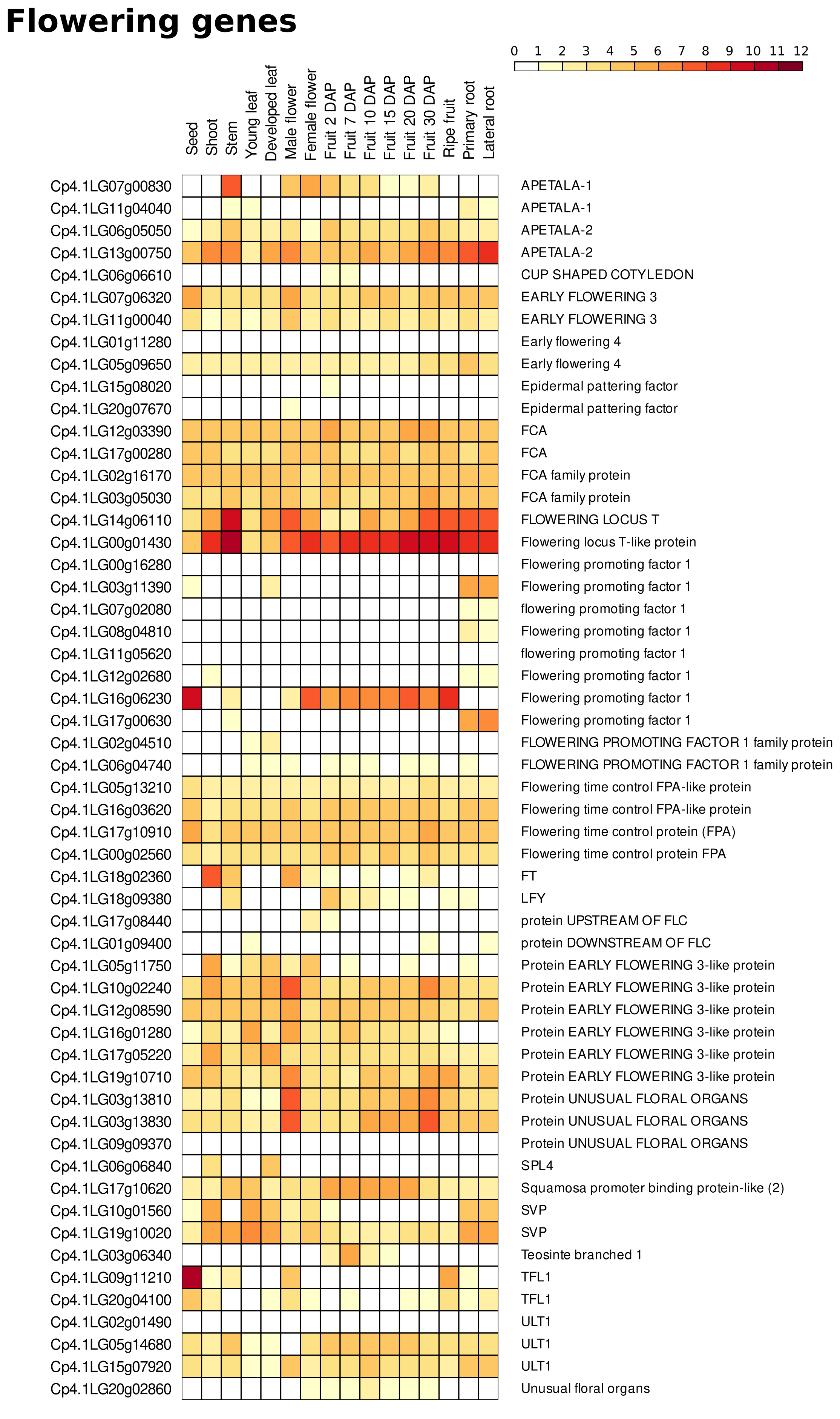

Supplement: Supplementary file 11 — Additional file 11. [file 12864_2021_7683_MOESM11_ESM.png]

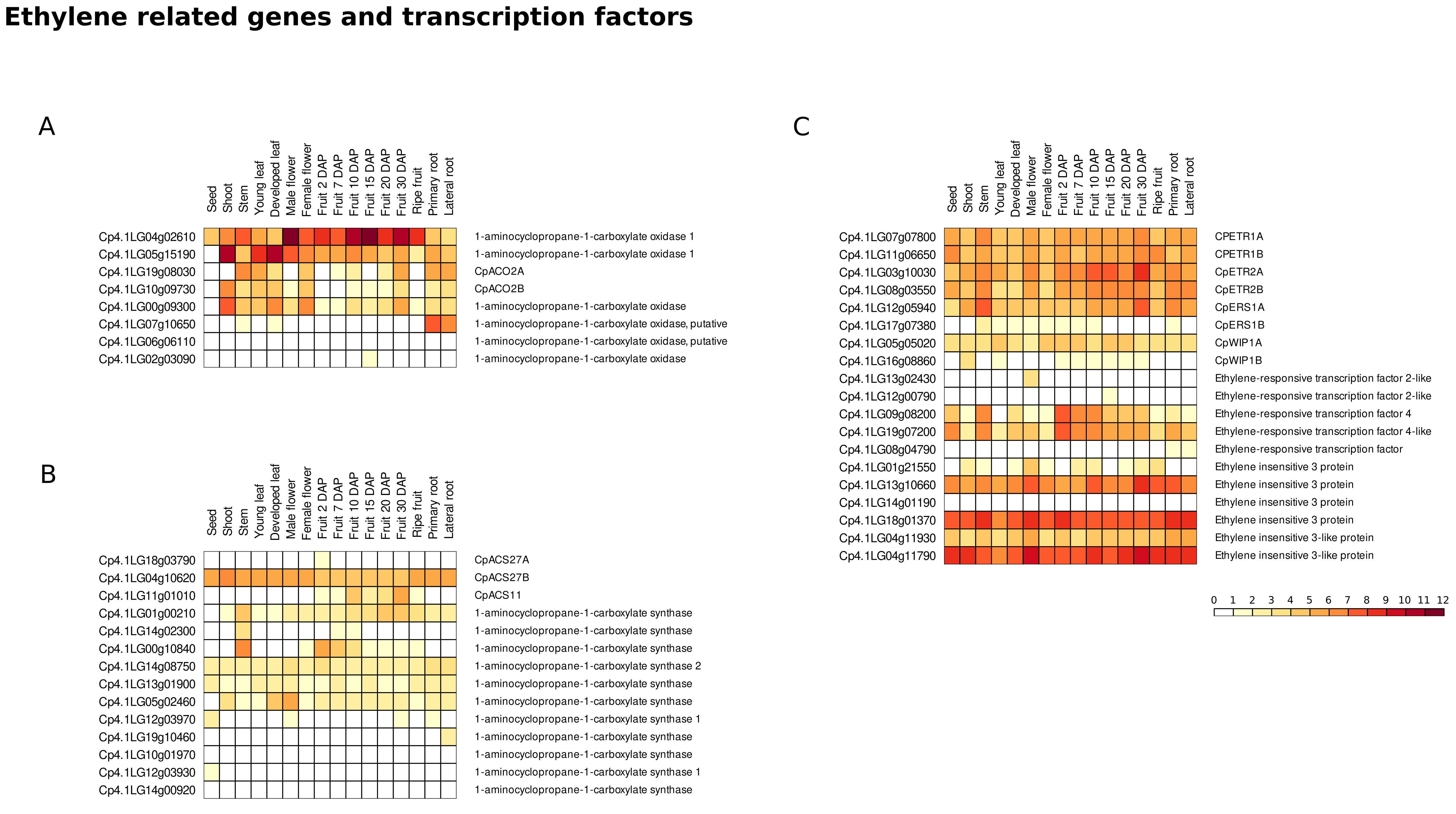

Supplement: Supplementary file 12 — Additional file 12. [file 12864_2021_7683_MOESM12_ESM.png]

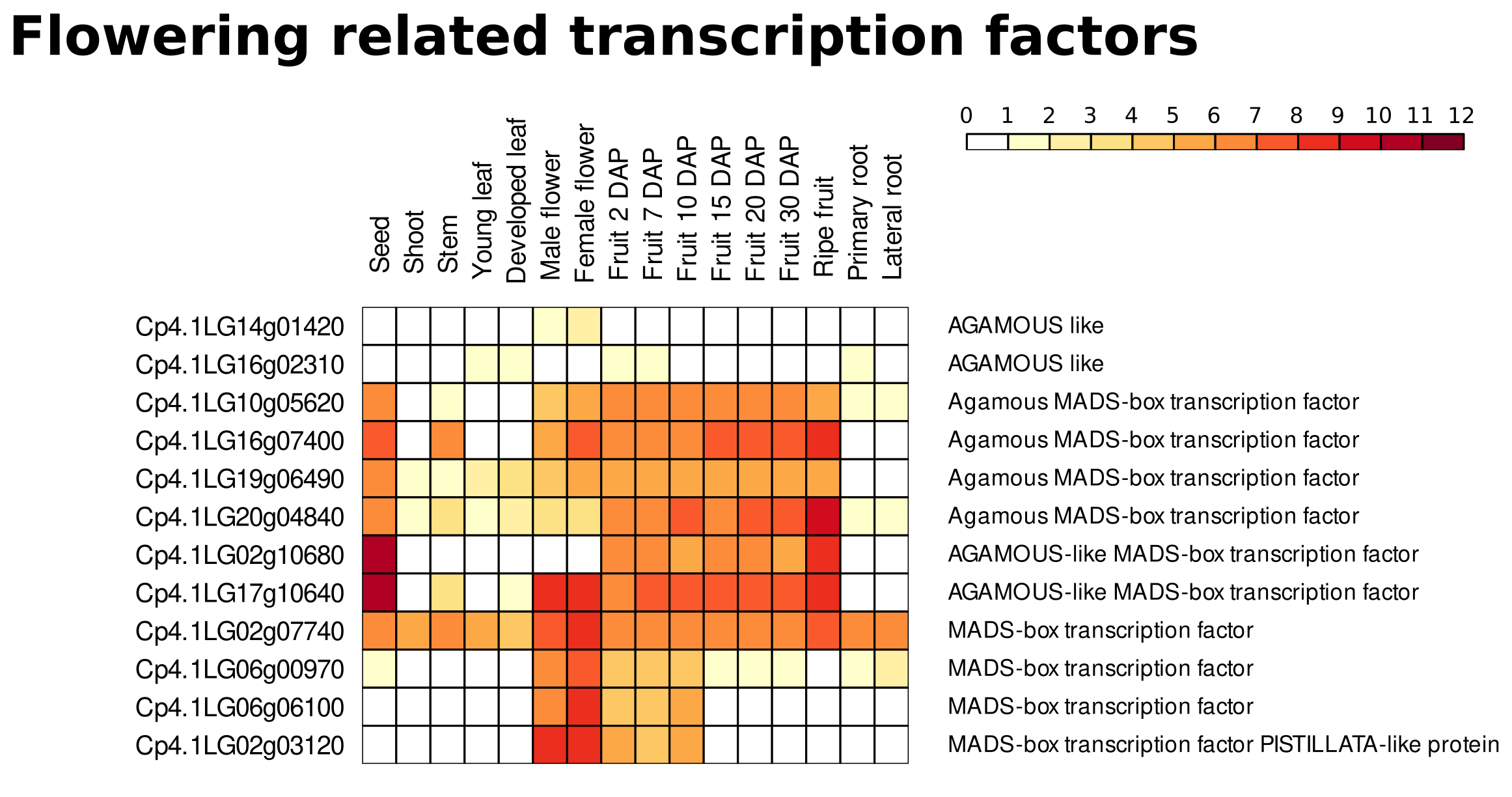

Supplement: Supplementary file 13 — Additional file 13. [file 12864_2021_7683_MOESM13_ESM.png]

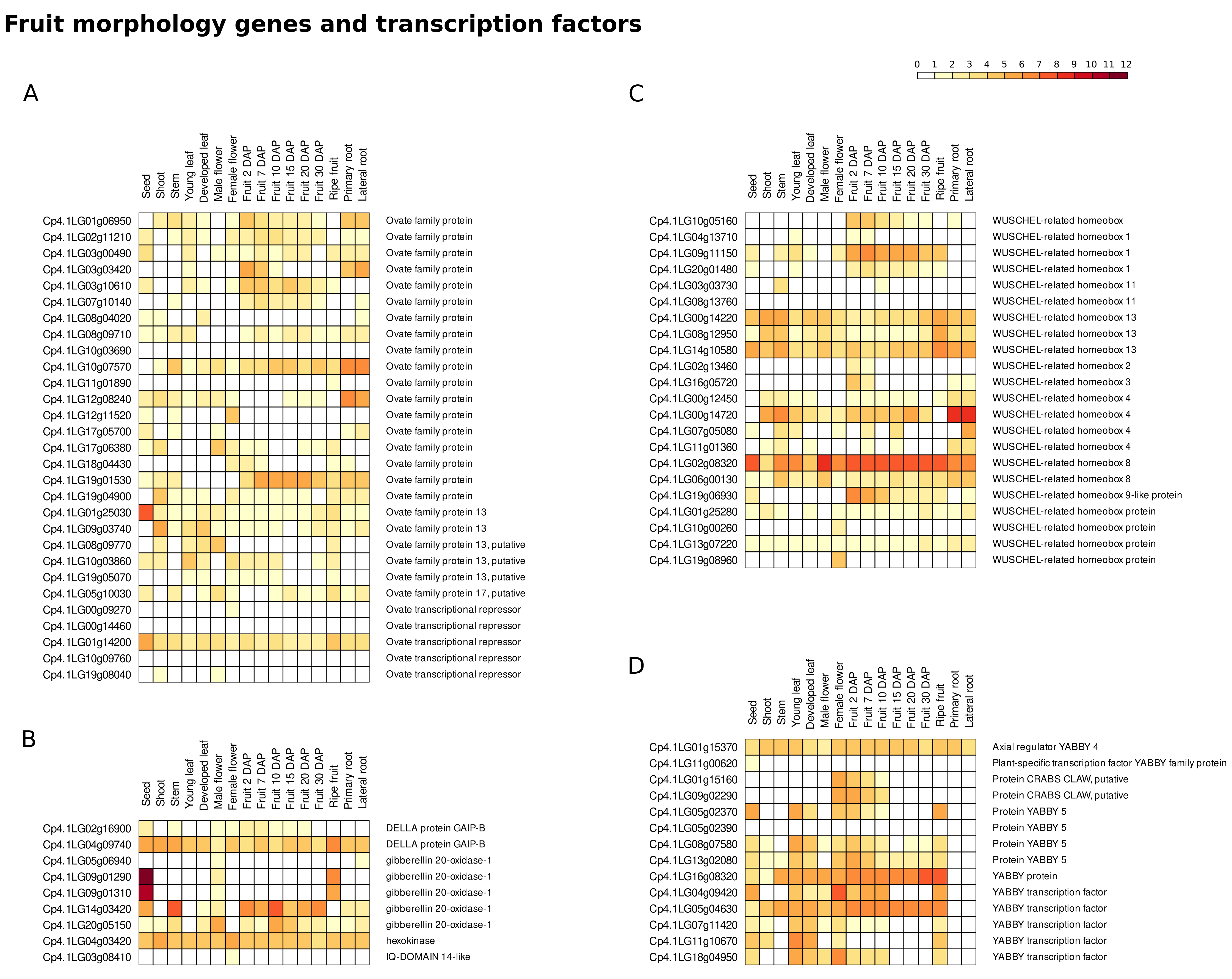

Supplement: Supplementary file 14 — Additional file 14. [file 12864_2021_7683_MOESM14_ESM.png]
